# Female sex and overweight are associated with a lower quality of life in patients with myasthenia gravis: a single center cohort study

**DOI:** 10.1186/s12883-023-03406-0

**Published:** 2023-10-10

**Authors:** Hannah Wilcke, Stefanie Glaubitz, Fabian Kück, Christoph Anten, David Liebetanz, Jens Schmidt, Jana Zschüntzsch

**Affiliations:** 1 Department of Neurology, University Medical Center, Göttingen, Germany; 2 Department of Medical Statistics, University Medical Center, Göttingen, Germany; 3 Department of Neurology and Pain Treatment, Immanuel Clinic Rüdersdorf, University Hospital of the Brandenburg Medical School Theodor Fontane, Rüdersdorf bei Berlin, Germany; 4 Faculty of Health Sciences Brandenburg, Brandenburg Medical School Theodor Fontane, Rüdersdorf bei Berlin, Germany

**Keywords:** Myasthenia gravis, Quality of life, Gender medicine, MG-ADL

## Abstract

**Background:**

Myasthenia gravis (MG) affects individuals as a chronic autoimmune disease for many years. Commonly, chronic diseases significantly reduce the patients’ quality of life. Aiming to improve the future quality of life in MG, this study assessed the factors impacting quality of life. As gender-specific medicine is becoming increasingly important, this study also focused on understanding gender differences in the outcome of MG.

**Methods:**

The study is a combined monocentric, retrospective and prospective database analysis of patient records based on 2,370 presentations of 165 patients with clinically, serologically and/or electrophysiologically confirmed MG over an observation period of up to 47 years. The data collection included the following parameters: antibody status, disease severity, age, medication use, gender, and disease duration. In addition, a prospective survey was conducted on the quality of life using the Myasthenia gravis-specific 15-item Quality of Life scale (MG-QoL15) and on the activities of daily living using the MG-specific Activities of Daily Living scale (MG-ADL).

**Results:**

Of the 165 patients, 85 were male (51.5%) and 80 were female (48.5%). The remaining baseline characteristics (e.g. age and antibody status) were consistent with other myasthenia gravis cohorts. A high body mass index (BMI) (p = 0.005) and a high disease severity (p < 0.001) were significantly associated with lower disease-specific quality of life. Additionally, the quality of life in women with MG was significantly reduced compared to male patients (19.7 vs. 13.0 points in the MG-QoL15, p = 0.024). Gender differences were also observable in terms of the period between initial manifestation and initial diagnosis and women were significantly more impaired in their activities of daily living (MG-ADL) than men (4.8 vs. 3.0 points, p = 0.032).

**Conclusion:**

Women with MG had significantly poorer disease specific quality of life compared to men as well as patients with a higher BMI. In order to improve the quality of life, gender-specific medicine and further investigation regarding a modification of the quality of life by lowering the BMI are essential and necessary.

**Trial registration:**

Study approval by the Ethics Committee of the University Medical Center Göttingen was granted (number 6/5/18).

## Background

Autoimmune myasthenia gravis (MG) is a chronic neuromuscular disease. The global prevalence is 12.4 people per 100,000 [1] making MG a rare disease. MG can manifest at any age [2]. MG with early onset affects mainly women, whereas the proportion of men with late onset predominates [2–4]. Overall, more women than men develop the disease, the ratio being three to two [5].The common clinical symptom of MG is a premature, physical strain-dependent and fluctuating muscle weakness [6], which can be highly heterogeneous in individuals with regard to severity and muscle involvement. MG is caused by different autoantibodies (AB) against the postsynaptic membrane of the neuromuscular junction [7–11].

The treatment regimens usually contain a combination of acetylcholinesterase inhibitors and different immunosuppressant drugs depending on the disease severity and comorbidities (8, 12–14). Some patients may require intravenous immunoglobulins (IVIG) and plasmapheresis (15). Thymectomy is currently one of the regular treatments for thymic hyperplasia or thymoma (16). Recently, several new targeted immunotherapies for the treatment of refractory and/or “active” MG have been developed and partially entered the market [17–19]. It is noteworthy, that MG-specific Activities of Daily Living (MG-ADL) and quality of life measured by the Myasthenia gravis-specific 15-item Quality of Life revised scale (MG-QoL15r) were among the primary and secondary outcome measures in clinical trials for the complement inhibitors and neonatal Fc receptor (FcRn) inhibitors [20–22].

Recently, the examination of the quality of life has become increasingly important in medicine, particularly in the field of neurology [23]. Chronic diseases such as MG can affect the quality of life negatively. Thus, the recording of the quality of life can help to identify possible influences on it and can also be used as a measurement for therapeutic effects on disease progression [24]. Therefore, the MG-QoL15 is available for disease-specific assessment of quality of life in MG. It consists of 15 questions and includes the categories mobility, symptoms, general satisfaction, and psychological well-being [25]. Previous studies have unanimously concluded that the quality of life of patients with MG is impaired. Gender, impairment in activities of daily living, pre-existing conditions, and disease severity were identified as influencing factors for disease specific quality of life [26–29].

The aim of the study was to identify the risk factors associated with reduced quality of life in MG and to examine gender-specific differences in quality of life, as well as in disease progression.

## Methods

### Patients and study design

The study can be classified as a mixed prospective-retrospective study. All patients, who were treated for MG at the University Medical Center Göttingen (UMG) between their initial diagnosis and November 30, 2019, and who gave their written informed consent to participate in the study were included. All the methods and procedures carried out in this study were in accordance with relevant guidelines and regulation. Starting point of the database analysis was the initial diagnosis of MG or – if the diagnosis was not made at UMG – the initial presentation at the neuromuscular outpatient clinic. End point was the last presentation in the outpatient clinic until 11/30/2019.

The retrospective database analysis included general disease information as well as specific information on the disease course and progression and medical history. This information was extracted from physicians’ letters, results of serological examinations, results of myasthenia gravis-specific tests (e.g. Quantitative Myasthenia Gravis Score (QMG)), evaluations of electrophysiological examinations, radiological findings, and classifications according to the Myasthenia gravis Foundation Association (MGFA) classification. All available documents of a patient since the initial diagnosis until the end of this study (11/30/2019) were considered for the database analysis.

Assessment of the quality of life and impairments in activities of daily living was prospectively collected during the data collection period (02/01/2018–11/30/2019) using the MG-QoL15 and the Myasthenia gravis-specific Activities of Daily Living scale (MG-ADL). In this study, the German translation of the MG-QoL15 questionnaire according to Fitzthum et al. (2015) was used (30). The questionnaires were filled in either during a presentation at the Department of Neurology at UMG or after postal delivery in the patient’s home. The disease severity was analyzed with the help of the QMG. There have been various modifications of the QMG in the last decades and the observation period of this study was long. For harmonization, the QMG version according to Barohn et al. has been chosen [31]. In order to adapt for historically missing values and to compare the QMG score between the patients, in this study the sum of QMG was divided by the number of tests performed, resulting in a total score between 0 and 3 points [32]. Since MG-Qol-15 was standard in our clinic during the years studied, we used it instead of MG-Qol-15r.

### Statistical analyses

Statistical analyses were performed with the statistical software R (version 3.4.0; (R Core Team 2018)) using the R packages lmerTest (version 3.1.1 [33]) and lme4 (version 1.1.21 [34]) for the mixed linear models, and survival (version 3.2.7 [35]) for the Cox regression model. For all statistical analyses, the significance level was set at α = 5% (p ≤ 0.05). No correction for multiple testing was made because the present study was exploratory.

At the outset, descriptive data analysis was performed to highlight the characteristics of the patient cohort. In order to prevent a disproportionate influence of patients with many clinical presentations compared with patients with only a few presentations, the numerical data were always averaged per patient over the number of presentations. On this basis the mean, standard deviation, median and the minimum and maximum were calculated. The p-values for metric variables were derived using the Mann-Whitney *U* test. In order to calculate the p-values for categorical variables, Fisher’s exact test was applied. For thymic hyperplasia, thymomas, thymectomy, glucocorticoids, ciclosporine, cyclophosphamide and comorbidities we analyzed the number of patients with at least one reported occurrence during the patient’s observation period.

In a further step, correlations were tested using regression analyses. In order to include repeated measurements per patient into the evaluation, univariate and multivariate linear mixed models with a random intercept for each patient were fitted for this purpose. In the results section, the estimate and the standard error for each independent variable as well as for the intercept were given for the linear mixed models in addition to the p-value. The dependent variable MG-ADL was log(x + 1)-transformed for the regression analysis. For the analysis of association between BMI and the scores QMG, MG-QoL15 and MG-ADL, we faced the problem that there were only few cases where BMI and one of the scores were measured at the same day. Thus, we also considered BMI measurements from visits being closest to the day of the evaluations of the scores allowing a maximum time difference of one year.

As a sensitivity analysis we additionally included sex and age in all mixed linear models and obtained similar results.

In order to examine gender differences in the risk of myasthenic crisis Cox regression was used for the analysis of the time to the first crisis.

## Results

### Basic characteristics

The patient population consisted of a total of 165 individuals ranging from the age of 12 to 91 years throughout the entire observation period. 2,370 individual presentations were included in the database analysis. On average, patients were presented to the hospital 14 times during the study period, with a minimum of one and a maximum of 65 presentations per patient. The retrospective observation period per patient comprised up to 47 years. With regard to the prospective surveys of the MG-ADL, 82 questionnaires were completed and evaluated by 55 patients. In addition, 101 MG-QoL15 questionnaires from 64 patients were analyzed.

Over the entire observation period, the average mean age at the patients’ visit was 59.8 years. At first onset of the symptoms, the patients were on average 54.4 years old, and at the time of initial diagnosis, the average age was 55.4 years (Table 1). The majority of patients (42.4%) suffered from mild generalized disease (MGFA class II). The mean QMG score was 0.49 (SD 0.36), whereas the survey of disease-specific quality of life showed a mean score of 16.7 (SD 13.0) and of activities of daily living a mean score of 3.99 (SD 3.5). A summary of patients’ demographics, clinical status and disease activity is listed in Table 1 (see Table 1).

**Table 1:** Basis characteristics

Characteristic	value	Characteristic	value
**Gender **(Number of patients (%))		**Scores**	
Male	85 (51.5%)	**QMG modified** (value ± SD)	0.49 ± 0.4
Female	80 (48.5%)	Female	0.56 ± 0.4
**Age at symptom onset** (years ± SD)	54.4 ± 19.3	Male	0.42 ± 0.3
Female	50.1 ± 20.8	Missing data (number of patients)	0
Male	58.4 ± 17.0		
**Age at diagnosis** (years ± SD)	55.4 ± 18.9	**MG-ADL (**value ± SD)	3.99 ± 3.5
Female	51.9 ± 20.2	Female	4.8 ± 3.7
Male	58.7 ± 17.1	Male	3.0 ± 3.0
**BMI** (kg/m^2^ ± SD)	28.0 ± 5.5	Missing data (number of patients)	110
Female	27.0 ± 5.8		
Male	29.1 ± 5.0	**MG-QoL15** (value ± SD)	16.7 ± 13.0
Missing data (number of patients)	41	Female	19.7 ± 12.4
		Male	13.0 ± 13.0
**MGFA** (number of patients (%))		Missing data (number of patients)	101
I	18 (10.9%)		
II	70 (42.4%)	**Treatment** (number of patients (%))	
IIA	34 (20.6%)	Pyridostigmine	164 (99.4%)
IIB	14 (8.5%)	Pyridostigmine extended release	139 (84.2%)
Not class.	22(13.3%)	Glucocorticosteroids	143 (86.7%
III	31 (18.8%)	IVIG	44 (26.7%)
IIIA	17 (10.3%)	AZA	120 (72.7%)
IIIB	10 (6.1%)	MMF	47 (28.5%)
Not class.	4 (2.4%)	**Thymectomy** (number of patients (%))	52 (31.5%)
IV	27 (16.4%)		
IVA	2 (1.2%)	Thymoma	17 (37.8%)
IVB	24 (14.5%)	Thymic hyperplasia	12 (26.7%)
Not class.	1 (0.6%)	No pathology	16 (35.6)
V	19 (11.5%)	**Myasthenic crisis in the past**	
**Serological status** (number of patients (%))		(number of patients (%))	
		No	123 (74.5%)
Anti-AChR-AB (+) *(missing data n = 1)*	137 (83.5%)	Yes	42 (25.5%)
Anti-MuSK-AB (+) *(missing data n = 75)*	5 (5.6%)	- 1 crisis	29 (17.6%)
Anti-Titin-AB (+) *(missing data n = 63)*	36 (35.3%)	- 2 crisis	5 (3.0%)
Anti-LRP4-AB (+) *(missing data n = 161)*	0 (0%)	- > 2 crisis	8 (4.8%)

### Effects on MG-QoL15

**Fig. 1:**
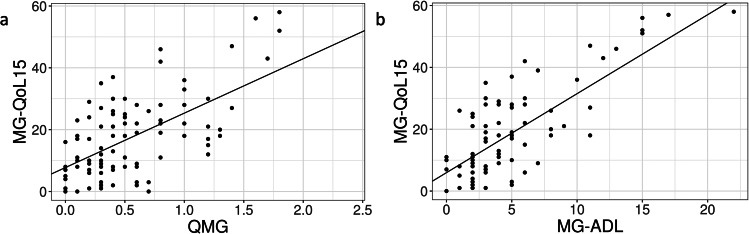
Correlations of quality of life and functional status. The linear mixed model shows a significant positive correlation between the QMG (modified) and the MG-QoL15 and the MG-ADL and the MG-QoL15. (**a**) Scatter-plot: significant positive correlation between QMG (modified) and MG-QoL15. A 1-point increase in QMG results in an estimated mean 17.6-point increase in MG-QoL15. Note: number of presentations: 99; number of patients: 63. Intercept: coefficient 7.75; SE 1.90; p < 0.001. QMG: coefficient 17.60; SE 2.80; p < 0.001. (**b**) Scatter-plot: significant positive correlation between MG-ADL and MG-QoL15. Note: number of presentations: 81; number of patients: 54. Intercept: coefficient 5.95; SE 1.76; p = 0.001. MG-ADL: coefficient 2.55; SE 0.30; p < 0.001 Abbreviations: MG-ADL = Myasthenia Gravis-Activities of Daily Living scale, MG-QoL15 = 15-item Myasthenia Gravis-Quality of Life scale, QMG = Quantitative Myasthenia Gravis Score

Linear mixed models identified a significant positive correlation between the QMG and the MG-QoL15: The higher the disease severity, the more severely the disease-specific quality of life was impaired. The estimated mean increase in the MG-QoL15 per one point increase in the QMG was 17.6 points (see Fig. 1a).

In addition, the extent of impairment in activities of daily living and the limitations in disease-specific quality of life correlated significantly positively with each other (see Fig. 1b).

A significant correlation was also detected between the QMG and the MG-ADL: The higher the severity of the disease, the more impaired were the patients’ MG-specific activities of daily living. An increase in disease severity (QMG) by one point led to an estimated increase in MG-ADL by the factor 2.7.

**Fig. 2:**
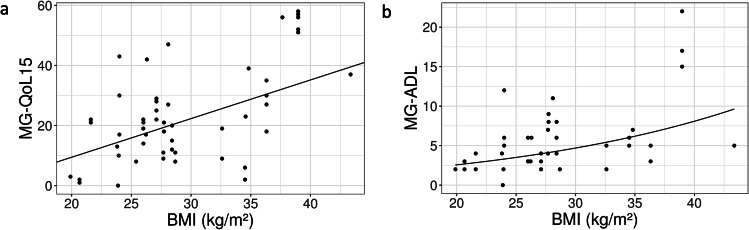
Correlation of BMI with quality of life and functional status. A higher BMI has a negative influence on quality of life as well as on functional disease specific status. (**a**) Scatter plot: significant positive correlation between BMI and MG-QoL15. Note: number of presentations: 53; number of patients: 27. Intercept: coefficient − 16.28; SE 12.01; p = 0.187. BMI: coefficient 1.29; SE 0.42; p = 0.005. (**b**) Scatter plot: significant positive correlation between BMI and MG-ADL. Note: number of presentations: 45; number of patients: 25. Intercept: coefficient 0.336; SE 0.527; p = 0.530. BMI: coefficient 0.05; SE 0.02; p = 0.017. The dependent variable MG-ADL was log(x + 1)-transformed Abbreviations: BMI = body mass index, MG-ADL = Myasthenia Gravis-Activities of Daily Living scale, MG-QoL15 = 15-item Myasthenia Gravis-Quality of Life scale

The BMI level also showed significant positive correlation with the degree of impairment of disease-specific quality of life and MG-specific activities of daily living (Fig. 2a and b). An increase in BMI by one point was associated with an estimated mean worsening of quality of life by 1.29 points or activities of daily living by about 5%. In contrast, no significant correlation between BMI and QMG could be found in the cohort (coefficient for BMI − 0.004, p = 0.414).

The MG-QoL15 score is not only related to the disease severity, activities of daily living and the BMI, but also correlates to the gender. Whereas the mean MG-QoL15 of the females was 19.68 points (SD 12.43, median 18.75), the mean MG-QoL15 of men was 13.00 points (SD 13.03, median 10.00). Thus, the mean MG-QoL15 of females was almost 6.7 points higher than that of males (p = 0.024).

Furthermore, a myasthenic crisis had significant negative influence on patients’ quality of life. Patients who had not experienced a myasthenic crisis – compared with those with having experienced a myasthenic crisis – stated a significantly better disease-specific quality of life and a significant lower impairment in MG-specific activities of daily living. After a myasthenic crisis, patients scored an average of 23.8 points on the MG-QoL15. In contrast, the MG-QoL15 of patients who had never experienced a myasthenic crisis averaged 14.5 points, meaning that they perceived their quality of life to be less impaired.

In order to consider a mutual influence of the parameters in the evaluation, the observation regarding the quality of life and the QMG was also carried out in a multivariate mixed model.

Analogous to the results of the univariate linear mixed model, disease severity correlated significantly positively with disease-specific quality of life in the multivariate model. Here, an one-point increase in QMG resulted in an estimated mean increase in MG-QoL15 of 16.92 points (see Table 2).

The occurrence of a myasthenic crisis did not significantly affect the MG-QoL15 score in multivariate analysis (see Table 2). Hence, the detected association between myasthenic crisis and poor quality of life is most probably due to the higher disease severity, expressed as QMG, after a myasthenic crisis and to the higher proportion of women experiencing a myasthenic crisis.

The MG-QoL15 score was not significantly influenced by the time between disease onset and initial diagnosis, and the time since initial diagnosis (see Table 2).

**Table 2:** Multivariate linear mixed model on factors influencing the MG-QoL15

	Coefficient	SE	p	
Experienced myasthenic crisis	0.04	3.23	0.989	
Patient age at disease onset	0.10	0.08	0.233	
Time between disease onset and initial diagnosis (in years)	0.25	0.32	0.452	
Time since initial diagnosis (in years)	-0.22	0.14	0.133	
Gender (female)	8.20	2.76	0.005	
QMG (modified)	15.52	2.98	< 0.001	

Note: Number of presentations: 98, number of patients: 62.

Intercept: Coefficient = 0.44, SE = 5.56, p = 0.937

Abbreviations: MG-QoL15 = 15-item Myasthenia Gravis-Quality of Life scale, QMG = Quantitative Myasthenia Gravis Score

### Gender differences

There were not only found significant repercussions on the female gender’s quality of life, but also evidence for additional differences between men and women in disease progression.

**Fig. 3:**
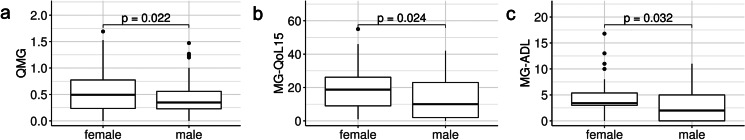
Gender differences regarding functional status and quality of life. The comparison of gender differences shows a significant higher disease activity, more strongly impaired functional status and a lower quality of life in women. (**a**) Boxplot on gender differences in QMG (modified). Note: women n = 80; mean 0.56; SD 0.40; median 0.49; [Min; Max] [0; 1.69]. Men n = 85; mean 0.42; SD 0.30; median 0.35; [Min; Max] [0; 1.47]. (**b**) Boxplot on gender differences in MG-QoL15. Note: women n = 35; mean 19.68; SD 12.43; median 10.00; [Min; Max] [1; 55]. Men n = 29; mean 13.00; SD 13.00; median 18.75; [Min; Max] [0; 42]. (**c**) Boxplot on gender differences in MG-ADL Note: women n = 30; mean 4.80; SD 3.74; median 3.41; [Min; Max] [0; 16.8]. Men n = 25; mean 3.01; SD 2.99; median 2.00; [Min; Max] [0; 11.0] Abbreviations: QMG = Quantitative Myasthenia Gravis Score, MG-QoL15 = 15-item Myasthenia Gravis-Quality of Life scale, MG-ADL = Myasthenia Gravis-Activities of Daily Living scale

At disease onset and initial diagnosis, females were significantly younger than males (median 53 vs. 63 years, p = 0.036; median 56 vs. 63 years, p = 0.011, respectively). In women, the time between disease onset and initial diagnosis was significantly longer (mean 2.15 vs. 0.87 years, median 0.5 vs. 0.2 years, p = 0.034). Moreover, women were affected more severely. Thus, the QMG as well as the MG-ADL were significantly higher in women (Fig. 3a-c).

With regard to thymic pathologies, gender-specific differences were also found. While thymic hyperplasia occurred more frequently in women (32% vs. 20%, missing data 120), thymomas were more frequently detected in men (45% vs. 32%, missing data 120). However, these differences were statistically not significant (p = 0.502 and p = 0.537, respectively). In contrast, the number of thymectomies performed differed significantly between the sexes. While 40% of women underwent thymectomy, only 23.5% of men underwent thymectomy (p = 0.029).

All but one patient in the cohort (n = 164, 99.4%) took pyridostigmine at least once during the observation period. While women were less likely to be treated with glucocorticoids than men (80% vs. 92.9%, p = 0.015), they were significantly more likely to receive IVIG (35% vs. 18.8%, p = 0.019). In contrast, the dose of pyridostigmine and of glucocorticoids did not differ significantly between genders (pyridostigmine: median 180.0 for both, p = 0.734; glucocorticoids: median 20.0 for women and median 17.6 for men, p = 0.717). Ciclosporine and cyclophosphamide were taken only by male patients, whereas only female patients were treated with tacrolimus and eculizumab.

Regarding concomitant diseases, female patients were significantly more frequently affected by depression (f = 28.7% vs. m = 15.3%, p = 0.040), autoimmune diseases (f = 27.5% vs. m = 9.4%, p = 0.004), in particular hypo-/hyperthyroidism and neurological diseases, than male patients. In contrast, men were significantly more likely to be affected by concomitant cardiovascular disease (f = 56.2% vs. m = 76.5%, p = 0.008).

The risk of myasthenic crisis was not significantly increased for females compared to males in univariate Cox regression analysis (hazard ratio 1.6, p = 0.145).

## Discussion

It is well-known that chronic diseases such as multiple sclerosis or amyotrophic lateral sclerosis have a negative impact on a patient’s quality of life [36–40]. In our study, we identified the quality of life – measured prospectively by the MG-QoL15 – in patients with MG to be impaired. One of the negative influencing factors is a high disease severity measured by the QMG. Studies that used the SF-36 questionnaire instead of the MG-QoL15 [41] or the MG composite score instead of the QMG also found a significant correlation between the quality of life and the disease severity [42, 43]. Another factor influencing disease-specific quality of life was the degree of limitation in activities of daily living, as measured by the MG-ADL. The higher the MG-ADL was in the present cohort, the more restricted was the disease-specific quality of life. These results are also consistent with those of other studies [28, 29, 44].

Based on the results of the present study, it can be concluded that severe myasthenic symptomatology measured by objective (e.g. QMG) or subjective (e.g. MG-ADL) parameters has a negative impact on the disease-specific quality of life. Therefore, adequate therapy appears to be essential not only to minimize the physical limitations caused by the disease, but also to improve the patients’ quality of life. Mendoza et al. (2020) found that a score of ≤ 8 points in the MG-QoL15 is – on average – considered acceptable by patients [45]. Since both in the present study and in the majority of studies available in the literature the average MG-QoL15 is considerably higher than this value [26–28, 46, 47], it seems that a relevant proportion of patients with MG receiving the therapeutic options available was not sufficient controlled to achieve a satisfactory quality of life [48].

Moreover, patients with MG are more frequently obese compared to the normal population [49], caused by low physical activity due to muscle weakness and therapy side effects (esp. by glucocorticoids) [50]. The average BMI of the cohort studied was also higher than the average BMI of the German population [51]. A high BMI may negatively affect the quality of life of patients with MG [39, 46]. The association of a high BMI with worse disease-specific quality of life as well as with MG-specific activities of daily living could be verified in our cohort.

Analogous to previous studies [48, 52, 53], women in the studied cohort were significantly more impaired in their disease-specific quality of life and in their disease-specific activities of daily living compared with men. Furthermore, the disease severity of the women was higher compared with the men. Additionally, the time between disease onset and initial diagnosis was significantly longer in women than in men. One reason for the higher disease severity in women or the higher impairment in MG-ADL could be hormonal influences. Previous studies described that sex hormones can influence antibody production in patients with MG. Interacting with the autoimmune regulator of the thymus, testosterone and estrogen may influence the production of anti-AChR-AB and thus have a crucial impact on the development and severity of MG [54]. The hypothesis that hormonal influences play a crucial role in disease progression is reinforced by the fact that postpartum women are at increased risk for the onset or worsening of MG [55]. In addition, exacerbations of the disease occur more frequently in women in association with menstruation [56]. The gender differences in pathophysiology could also lead to unequal efficacy of disease-specific medications in men and women. In the present study, significant gender differences in QMG and MG-ADL occurred despite comparable ingested doses of disease-specific medication.

As already mentioned, the study showed longer time elapses between the first symptoms and diagnosis in women. The longer period of uncertainty and the increased suffering associated with this can have a negative effect and can cause a later start of therapy. The delay in initiating targeted therapy may explain the greater severity of the disease in women. The significantly higher proportion of female patients receiving IVIG therapy also supports the observation that the severity is higher in women. In addition, the side effect profile of glucocorticoid medication seems to be more pronounced in women [57], which may explain both the reduced quality of life and the frequency of therapy escalation to IVIGs.

There are limitations of the study, because – due to its retrospective study design – data on certain characteristics, such as antibody status or concomitant diseases, could not be found in the files of some patients. Nevertheless, the overall willingness to participate in the prospective part of the study was low. The monocentric approach must also be regarded as a limitation. Furthermore, concerning the analysis of the influence of the BMI on the quality of life and the functional status, reverse causality cannot be ruled out, and our results rely on the assumption that BMI changes over short time periods are negligible.

However, we clearly show that the time to diagnosis needs to be shortened, especially with respect to gender aspect, and that the use of patient reported outcome is an important tool in the definition of treatment goals. Complimentary, lifestyle counselling should be provided. This means that treatment goals for MG patients will become even more patient-centered as has already been incorporated in the newly published guidelines of the German Society for Neurology [58].

## Conclusion

Despite many available drug treatment options, the results of the study confirm that patients with MG are limited in their quality of life. Since the quality of life is significantly influenced by disease severity, the development of new therapeutic approaches represents an important element in improving the quality of life. In light of approval of new therapies such as FcRn inhibitors and complement inhibitors, MG patient care is currently entering a phase of paradigm shift [20, 59–61]. To meet the new therapeutic concepts, e.g. “hit hard and early” and “minimal symptom expression”, the need for a timely diagnosis has to be emphasized, especially for women who are typically faced with a prolonged diagnosis. Our data underscore the need to conduct further studies that aim to delineate reasons for gender differences and how to overcome them.

Furthermore, patients with myasthenia gravis should be informed that a high BMI can have a negative impact on their disease-specific quality of life. With a holistic view to future, dietary interventions, given an appropriate benefit/risk ratio, are an interesting option for further studies involving patients in the design from the beginning.

## Data Availability

data are available on request from the corresponding author.

## Abbreviations

AB: Antibody
AChR: Acetylcholine receptor
AZA: Azathioprine
BMI: Body mass index
F: Female
FcRn: Neonatal Fc receptor
IVIG: Intravenous immunoglobulin
LRP4: Lipoprotein receptor-related protein-4
M: Male
MG-ADL: Myasthenia gravis-specific Activities of Daily Living scale
MG-QoL15: Myasthenia gravis-specific 15-item Quality of Life scale
MG-QoL15r: Myasthenia gravis-specific 15-item Quality of Life revised scale
MG-QoL60: Myasthenia gravis-specific 60-item Quality of Life scale
MG: Myasthenia gravis
MGFA: Myasthenia gravis Foundation Association
MMF: Mycophenolate mofetil
MuSK: Muscle-specific kinase
QMG: Quantitative Myasthenia Gravis Score
SD: Standard deviation
SE: Standard error
SF-36: Short form-36
UMG: University Medical Center Göttingen
